# METTL14 enhances the m6A modification level of lncRNA MSTRG.292666.16 to promote the progression of non-small cell lung cancer

**DOI:** 10.1186/s12935-024-03250-3

**Published:** 2024-02-07

**Authors:** Xianxiu Ji, Xiaoying Wan, Hui Sun, Qinfang Deng, Shuyan Meng, Boxiong Xie, Songwen Zhou

**Affiliations:** 1 Department of Oncology, Shanghai Pulmonary Hospital, Tongji University School of Medicine, No.507, Zhengmin Road, Yangpu District, Shanghai, 200433 China; 2 Department of Thoracic, Shanghai Pulmonary Hospital, Tongji University School of Medicine, Shanghai, 200433 China

**Keywords:** Non-small cell lung cancer, A549 cells, METTL14, m6A modification, lncRNA MSTRG.292666.16

## Abstract

**Background:**

m6A modification has close connection with the occurrence, development, and prognosis of tumors. This study aimed to explore the roles of m6A modification and its related mechanisms in non-small cell lung cancer (NSCLC).

**Methods:**

NSCLC tissues and their corresponding para-cancerous tissues were collected to determine the m6A levels of total RNA/lncRNAs and the expression of m6A modification-related genes/lncRNAs. Then, A549 cells were transfected with si-METTL14 or oe-METTL14, and the cell transfection efficiency was assessed. Subsequently, the viability, apoptosis, cell colony formation, migration and invasion of the different cells were determined. Finally, the nude mouse tumorigenicity experiments were performed to observe the effects of METTL14 in vivo.

**Results:**

Compared to the para-NSCLC tissues, the m6A level and METTL14 expression were both significantly increased in the NSCLC tissues (*P* < 0.05). Based on the expression of METTL14 in the different cell lines, A549 cells were chosen for further experiments. Then, the A549 cells with METTL14 knockdown and overexpression were successfully established, as well as it was found that METTL14 knockdown could inhibit the viability, colony formation, migration, and invasion of A549 cells, while facilitate their apoptosis. In vivo experiments also showed that METTL14 knockdown could inhibit tumor formation and growth. Additionally, the m6A level of MSTRG.292666.16 was higher in the NSCLC tissues; and after METTL14 knockdown, the expression and m6A level of MSTRG.292666.16 were both significantly reduced in A549 cells, and vice versa.

**Conclusion:**

METTL14 may promote the progression of NSCLC through up-regulating MSTRG.292666.16 and enhance its m6A modification level.

## Introduction

In 2022, the global mortality rate of lung cancer still ranks first among the top 10 tumors [1]. Non-small cell lung cancer (NSCLC) accounts for about 80-85% of lung cancer, has higher heterogeneity and invasiveness [2]. The next-generation sequencing analysis shows that NSCLC is often accompanied by gene mutations, and it is easy to obtain drug resistance after single targeted therapy, chemotherapy, or immunotherapy, resulting in distant metastasis of cancer cells, and leading to disease progression and even death [3, 4]. With the widespread development of precision medicine, molecular typing of NSCLC has been carried out to the progression free survival (PFS) and overall survival (OS) of patients through personalized comprehensive treatment [5, 6]. However, distant metastasis and postoperative metastasis in drug-resistant lung cancer seriously affects the therapeutic effectiveness, which is a major clinical challenge [7]. At present, the 5-year survival rate of advanced NSCLC is only 15%, and metastasis of important organs such as the brain, bone, and liver seriously affect survival; as well as 21.7% of patients with stage I lung cancer will experience recurrence and metastasis after radical surgery [8, 9]. Therefore, it is urgent to conduct in-depth research on the potential mechanisms of NSCLC metastasis to unearth new key factors, and develop novel drugs with fewer side effects than chemotherapy and immunotherapy.

Abnormal RNA modification is involved in tumor development and metastasis, which is the currently research hotspot [10]. N6-methyladenosine (m6A) is the most abundant mRNA modification, which regulates gene expression by regulating RNA processing, localization, translation, and decay. The interference with m6A modification can lead to a variety of diseases, including malignant tumors [11]. A previous study has found that m6A levels of mRNA or total RNA in human gastric cancer (GC) tissues are significantly increased compared to the normal tissues, and METTL14 is a key regulator of m6A disorders in GC [12]. Another research showed that significantly reduced overall m6A level abundance was closely related to the poor prognosis of patients with bladder cancer (BC), and FTO could promote tumorigenesis of BC via the MALAT/miR-384/MAL2 axis in m6A RNA modification manner [13]. These reports indicated that m6A modification level has close connection with the occurrence, development, and prognosis of tumors, but its role and correlation in NSCLC remain unclear.

Long non-coding RNAs (lncRNAs), a kind of RNA molecules with the length of more than 200nt, lack protein-coding capabilities, and can regulate gene expression by interacting with proteins, RNA, and DNA [14]. More and more evidence has shown that lncRNAs play cancer-promoting and cancer-inhibiting roles in the occurrence and development of tumors, as well as participate in the processes of tumor growth, infiltration, and metastasis [15, 16]. In addition, the roles of m6A modification of lncRNAs in tumors is gradually being revealed. For example, there were 7 m6A modification sites on lncRNA FEZF1-AS1, and silencing METTL14, METTL3, YTHDF1, and YTHDF2 could reduce the FEZF1-AS1 levels; as well as knocking out FEZF1-AS1 could inhibit the proliferation, migration, and invasion of NSCLC cell lines [17]. These implied that m6A methylation of lncRNA FEZF1-AS1 may exert a “carcinogenic” effect on NSCLC through regulating METTL14, METTL3, YTHDF1 and YTHDF2. Wen et al. [18] demonstrated that there was 4 credible m6A modification site on lncRNA NEAT1-1, as well as high m6A modification level was associated with bone metastasis of prostate cancer, and m6A modification of NEAT1-1 was a strong predictor of eventual death. Our previous investigation found that lncRNAs MSTRG.292666.16 and MSTRG.292667.12 were both significantly up-regulated in osimertinib-resistant plasma compared to the osimertinib-sensitive plasma from NSCLC patients, which may be closely connected with the drug resistance and metastasis of NSCLC [19]. However, the function and molecular mechanisms of m6A-modified lncRNAs MSTRG.292666.16 and MSTRG.292667.12 in NSCLC development still require further research and clarification.

In this study, NSCLC tissues and their corresponding paracancerous tissues were collected to determine the m6A levels, and the expression of m6A modification-related genes. Then, METTL14, a kind of RNA methyltransferase complexes, was chosen to explore its effects on NSCLC progression in vitro. Additionally, the expression and m6A levels of the two related lncRNAs were also measured, and MSTRG.292666.16 was selected as the objective. Finally, the relationship between METTL14 and MSTRG.292666.16, and the effects of METTL14 on tumor growth in vivo were studied. This work will improve our understanding of the potential functions of m6A modifications in NSCLC development, and provide novel targets for the management of NSCLC.

## Materials and methods

### Sample collection and cell culture

From January to December 2022, nine NSCLC patients were recruited from Shanghai Pulmonary Hospital, and all cases were confirmed by pathological examination. Then, the fresh cancer tissues were obtained from all the patients, and their corresponding para-carcinoma tissues were also collected as the controls. The research protocol was approved by the Ethics Committee of Shanghai Pulmonary Hospital, Tongji University School of Medicine (approval no. K23-070Y), and informed consent was obtained from all participants.

Human NSCLC cell lines, including NCI-H1650 cells, NCI-H1299 cells, and A549 cells, as well as human bronchial epithelial cell line BEAS-2B cells were all purchased from Cell bank, Chinese Academy of Sciences (Shanghai, China). The cells of NCI-H1650 and NCI-H1299 were cultured in RPMI 1640 medium (cat. no. 11,875,093, Thermo Fisher Scientific, Waltham, MA, USA) supplemented with 10% fetal bovine serum (FBS, cat. no. A5669701, Thermo Fisher Scientific) and 100 U/mL penicillin and 100 g/L streptomycin (cat. no. 15,140,148, Thermo Fisher Scientific); whereas the A549 cells were cultured in F-12 K medium (cat. no. 21,127,022, Thermo Fisher Scientific, Waltham) containing 10% FBS and 100 U/mL penicillin and 100 g/L streptomycin. Additionally, the BEAS-2B cells were cultured in BEGM BulletKit medium (cat. no. CC-3170, LONZA, Basel, Switzerland). All the cell lines were maintained in an incubator with 5% CO_2_ at 37 ℃.

### Detection of m6A methylation in tissue samples

Total RNA was isolated from the cancer tissues and their corresponding para-carcinoma tissues using RNAiso Plus kit (Trizol, Takara Biomedical Technology Co., Ltd., Beijing, China) according to the manufacturer’s protocols. The purity and concentration of total RNA were detected using a microplate reader (Thermo Fisher Scientific). Then, a m6A RNA Methylation Quantification Kit (Epigentek Group Inc., Farmingdale, NY, USA) was employed to measure the levels of RNA m6A methylation in the NSCLC tissue samples in accordance with the recommendations of the manufacturer.

### Real-time quantitative PCR (RT-qPCR)

RNAiso Plus kit (Trizol, Takara) was used to extract the total RNA from the cancer tissues and cells. Then, based on the instructions of the manufacturer, the extracted total RNA was reverse transcribed into cDNA using PrimeScriptTM II 1st Strand cDNA synthesis Kit (Takara). The temperature protocol of reserve transcription was 37 °C for 60 min and 85 °C for 5s. The primers were synthesized and provided by Shanghai Sangon Biotech (Shanghai, China), and the sequences of all primers were shown in Table 1. The qPCR reaction was initiated at 50 °C for 2 min, a total of 40 cycles at 95 °C for 2 min, 95 °C for 15 s, and at 60 °C for 60 s, and then melted at 95 °C for 15 s, 60 °C for 60 s, and 95 °C for 15 s. *GAPDH* served as the reference gene, and the relative mRNA expression levels of *METTL14*, *METTL3*, *WTAP*, *FTO*, lncRNA MSTRG.292666.16, and lncRNA MSTRG.292667.12 were analyzed using the 2^−ΔΔCT^ method.

**Table 1:** Sequences of all primers

Primer	Sequences (5’-3’)
METTL14	F: AGTGCCGACAGCATTGGTG
	R: GGAGCAGAGGTATCATAGGAAGC
METTL3	F: TTGTCTCCAACCTTCCGTAGT
	R: CCAGATCAGAGAGGTGGTGTAG
WTAP	F: CTTCCCAAGAAGGTTCGATTGA
	R: TCAGACTCTCTTAGGCCAGTTAC
FTO	F: ACTTGGCTCCCTTATCTGACC
	R: TGTGCAGTGTGAGAAAGGCTT
MSTRG.292666.16	F: CTGGAGTGCAGTGGCTATTC
	R: AGGCTGAGGTGGGAGGAT
MSTRG.292667.12	F: AGGCTGAGGTGGGAGGAT
	R: CTGGGCAACATAGCGAGAC
GAPDH	F: TGACAACTTTGGTATCGTGGAAGG
	R: AGGCAGGGATGATGTTCTGGAGAG

### Western blot

Total protein was isolated from the tissue samples and different cells using RIPA lysis buffer (Beyotime Biotechnology, Shanghai, China), and then a BCA assay kit (Boster Biological Technology Co. LTD, Wuhan, China) was utilized to examine the concentrations of the isolated total protein. After that, the total protein samples (20 μg) were separated by SDS-PAGE, transferred to PVDF membranes, and blocked with 5% skim milk. After blocked at 37 °C for 1 h, the membranes were incubated with the anti-METTL14 antibody (1:2000, Proteintech Group, Inc.), and anti-GAPDH antibody (1:10000, Proteintech Group, Inc.) at 4 °C overnight. After washing, the membranes were incubated with the secondary antibodies (1:5000, 1:5000, Jackson ImmunoResearch) at 37 °C for 2 h. After washing, the protein bands were visualized using an ECL assay kit (Beyotime Biotechnology) and a chemiluminescence apparatus (Shanghai Tanon Science & Technology Co., Ltd.).

### Immunohistochemical (IHC) staining

The cancer tissues and their corresponding para-carcinoma tissues were fixed with 4% paraformaldehyde for 24 h, and after washing with distilled water for 30 min, the tissue samples were used for dehydration (70% ethyl alcohol for 3 h, 80% ethyl alcohol for 45 min, 95% ethyl alcohol I for 50 min, 95% ethyl alcohol II for 50 min, 100% ethyl alcohol I for 40 min, 100% ethyl alcohol II for 40 min, xylene I for 1 h, and xylene II for 1 h), waxdip, embedding, and cutting into 4-μm slices. Subsequently, the slices were baked at 60 °C for 30 min, and then were performed for dewaxing, rehydration, antigen retrieval, and closure of endogenous peroxides. Then, the slices were incubated with the anti-METTL14 antibody (1:250, Proteintech Group, Inc.) at 4 °C overnight. On the next day, the slices were first incubated at room temperature for 30 min, and after washing, the slices were incubated with the horseradish enzyme labeled secondary antibody (1:200, Jackson ImmunoResearch) at 37 °C for 30 min. After washing with PBS for three times, diaminobenzidine was added for 1 min, and then the slices were stained with hematoxylin for 30 s. After dehydration, the slices were sealed with neutral resins, and images were taken under a microscopy.

### Cell transfection

The si-negative control (si-NC), and si-METTL14-1/2/3 were designed and synthesized by GENEray Biotechnology (Shanghai, China), as well as the pcDNA3.1(+)-METTL14 plasmid (oe-METTL14) and pcDNA3.1(+) plasmid (oe-NC) were prepared and purchased from Sangon Biotech (Shanghai, China). The methods of cell transfection were described as previously [20]. Briefly, the A549 cells in good condition were inoculated into a 24-well plate at a density of 4 × 10^4^ cells/well, and cultured overnight. On the next day, the cell medium was changed to the serum-free medium. The A549 cells were transfected with 15 pmol si-NC or si-METTL14-1/2/3, and 1 μg pcDNA3.1(+)-METTL14 plasmid or pcDNA3.1(+) plasmid using Lipofectamine 200 (Thermo Fisher Scientific) based on the protocols of the manufacturer. After 6 h of transfection, the medium was replaced with the complete medium, and the cells continued to culture for 48 h. The total RNA was extracted from the cells with different transfection, and the expression of METTL14 was determined by western blot to assess the cell transfection efficiency.

### Cell viability, apoptosis, and colony formation assays

The viability of A549 cells with different transfections were determined using Cell Counting Kit-8 (CCK-8, Beyotime Biotechnology). Briefly, the different A549 cells were collected, and seeded onto a 96-well plate at a density of 1 × 10^4^ cells/well. After cultured for another 24 h, 48 h, and 72 h, 10 μL of CCK-8 solution was added to each well, and incubated for 2 h. Then, a microplate reader was applied to measure the absorbance at 450 nm.

In accordance with the manufacturer’s instructions, Annexin V-FITC apoptosis assay kit (Beyotime Biotechnology) was applied to determine the cell apoptosis. Briefly, the cells with different treatment were harvested, and centrifuged at 1000 rpm for 5 min. Then, the sediments were resuspended with 195 μL Annexin V-FITC binding buffer, and extra 5 μL Annexin V-FITC was added. After slightly mixing, the cells were stained with 5 μL propidium iodide (PI), and maintained in the dark at room temperature for 20 min. Finally, a flow cytometer was applied to acquire the cells, and the total cell apoptosis rate was calculated.

In addition, the cell colony formation of the cells was also evaluated. The cells with different treatments were seeded to a 6-well plate at a density of 400 cells/well, and cultured in the incubator for 14 days. After the obvious cell colonies were visible to the naked eye, the supernatant was discarded, and the sediments were washed with PBS twice. Then, the cells were fixed with 4% paraformaldehyde at room temperature for 10 min, and stained with crystal violet at room temperature for 10 min. After removing the excess crystal violet, and washed with distilled water, the cell pictures were observed and photographed under a microscope.

### Cell migration and invasion assays

Transwell chambers (8-μm pore size; Guangzhou Jet Bio-Filtration Co. Ltd., Guangzhou, China) were used to evaluate the cell migration and invasion. For the determination of cell invasion, the Transwell chambers were pre-coated with a layer of Matrigel (excreta cellular matrix gel, Corning, NY, USA). Briefly, the cells with different transfections were obtained to inoculated to the upper insert of the chambers, and the lower chamber was the complete medium. After 48 h of incubation, the cells were washed with PBS twice, and fixed with 4% paraformaldehyde at room temperature for 20 min. After washing, 0.1% crystal violet was added to the cells, and incubated at room temperature for 20 min. After removing the excess dye and air drying, the cell images were observed under an inverted microscope, and the relative cell number was analyzed.

### Methylated RNA immunoprecipitation (MeRIP)-qPCR

The m6A methylation levels of lncRNA MSTRG.292666.16 were determined by MeRIP-qPCR. Briefly, the total RNA in the cells with different treatments was extracted, and then poly(A)RNA was specially captured from50 μg total RNA using Dynabeads Oligo (dT) (Thermo Fisher Scientific) by two rounds of purification. Then, the captured RNA was fragmented into small pieces using Magnesium RNA Fragmentation Module (NEB, USA) UNDER 86 °C for7 min. The cleaved RNA fragments were incubated with m6A-specific antibody (Synaptic Systems, Germany) in IP buffer (50 mM Tris-HCl, 750 mM NaCl, and 0.5% Igepal). The IP RNA was reverse-transcribed into cDNA by SuperScriptTM II Reverse Transcriptase (Thermo Fisher Scientific), and then m6A methylation levels of lncRNA MSTRG.292666.16 and the expression of MSTRG.292666.16 were determined by qPCR.

### Nude mouse tumorigenicity assay

Eighteen SPF BALB/C male nude mice aged 4–6 weeks were purchased from Shanghai SLAC Laboratory Animal Co., Ltd (Shanghai, China). All the mice were maintained under controlled temperature (24 ± 2 °C) and humidity (50 ± 5%) conditions, with a 12 h light/dark cycle, as well as were free access to food and water *ad libitum* during the experiment. After 7 days of acclimatization, the mice were randomly divided into three groups (*n* = 6 for each group): control, si-NC, and si-METTL14 groups. Before animal experiments, A549 cells with different treatments were harvested, and washed with PBS twice. Then, the serum-free medium was used to adjust the concentration of the cells to 5 × 10^7^ cells/mL. After that, the mice in the control, si-NC, and si-METTL14 groups were subcutaneously injected with 100 μL A549 cells, 100 μL A549 cells transfected with si-NC (5 × 10^7^ cells/mL), and 100 μL A549 cells transfected with si-METTL14 (5 × 10^7^ cells/mL) in the axilla of the right forelimb, respectively. After tumor grafting, the mental state, activity, diet, urine, and bowel movements of the nude mice were observed regularly every day. The longest and shortest diameters of subcutaneous graft were measured with vernier calipers every week, and the relevant data were recorded in time. After feeding for 5 weeks, the nude mice were killed by cervical dislocation, and the tumor was stripped, cleaned, and photographed. The formula of subcutaneous graft tumor volume (V) was calculated as follows: V (mm^3^) = longest diameter of tumor (mm) × the shortest diameter (mm) × the shortest diameter (mm) × 0.5. According to the obtained tumor volume, the growth curve of transplanted tumor was plotted. The animal experiments were conducted in consistent with the National Medical Advisory Committee (NMAC) guidelines, and were approved by the Institutional Animal Care and Use committee of Shanghai Pulmonary Hospital, Tongji University School of Medicine (approval no. K23-070Y).

### Statistical analysis

Data were expressed as mean ± standard deviation, and GraphPad prism 5 software (San Diego, CA, USA) was applied for data processing and statistical analyses. For the comparison between the two groups, unpaired t test was applied to calculate the differences, while the differences among multiple groups (≥ 3) was analyzed by one-way analysis of variance followed by Tukey’s test. A value of *P* < 0.05 was thought of statistically significance.

## Results

### The m6A methylation level and expression of methylation-related genes in the NSCLC tissue samples

**Fig. 1:**
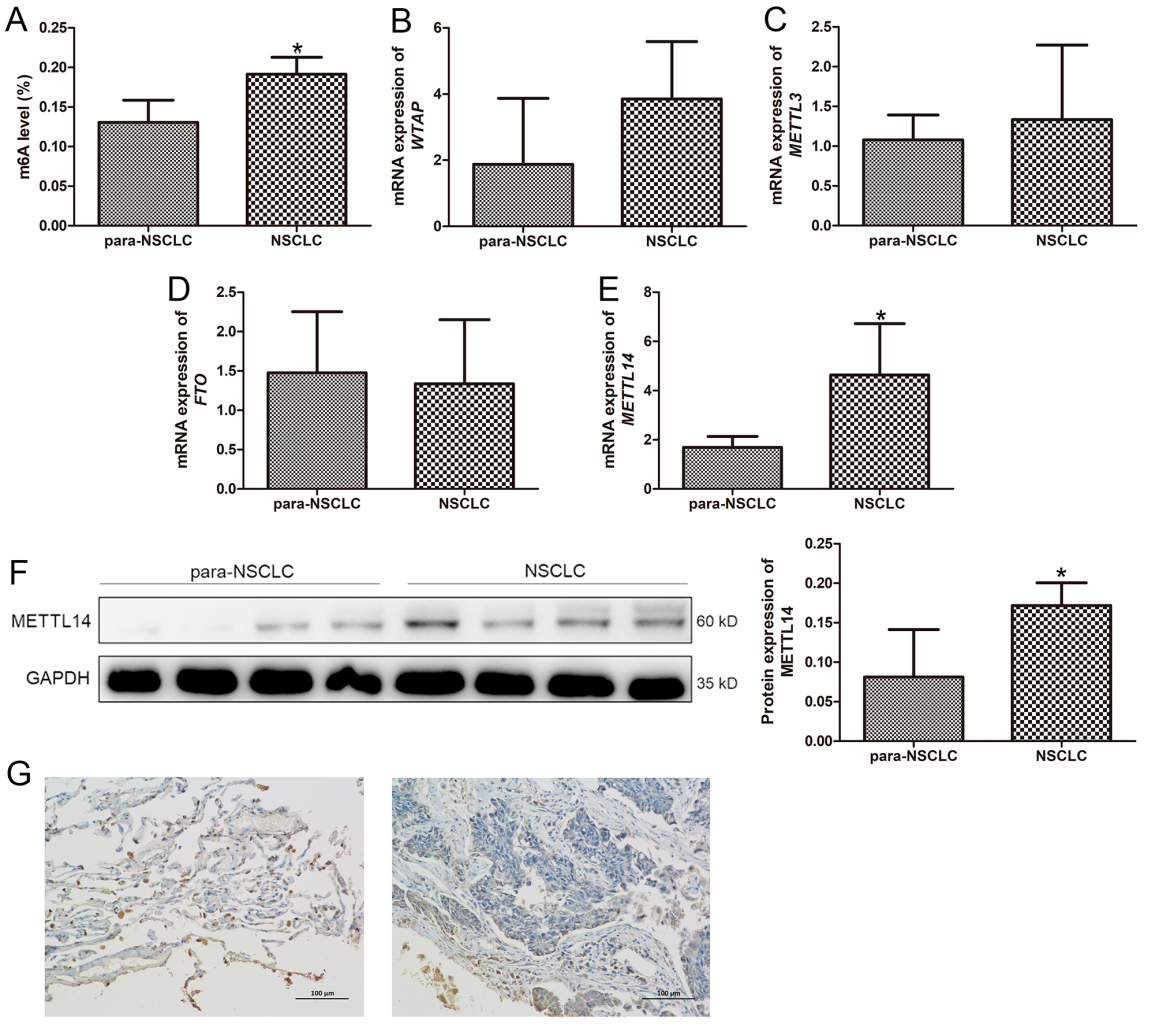
The m6A methylation level and expression of methylation-related genes in the non-small cell lung cancer (NSCLC) tissue samples. (**A**) The m6A methylation level in NSCLC. (**B**) The mRNA expression of *WTAP* in the NSCLC tissue samples. (**C**) The mRNA expression of *METTL3* in the NSCLC tissue samples. (**D**) The mRNA expression of *FTO* in the NSCLC tissue samples. (**E**) The mRNA expression of *METTL14* in the NSCLC tissue samples. (**F**) The protein expression of METTL14 in NSCLC using western blot. (**G**) The expression of METTL14 in NSCLC by immunohistochemical staining. * *P* < 0.05, compared with the para-NSCLC

In order to understand the m6A methylation in NSCLC, we determined the m6A methylation level and expression of methylation-related genes in the NSCLC tissue samples. It was found that compared with the para-NSCLC tissue samples, the m6A level in the NSCLC tissue samples was significantly higher (*P* < 0.05, Fig. 1A). Then, the expression of methylation-related genes, including *WTAP*, *METTL3*, *FTO* and *METTL14*, were measured by RT-qPCR. It is clear that there was no significant difference in mRNA expression of *WTAP*, *METTL3*, and *FTO* between the para-NSCLC tissues and NSCLC tissues (*P* > 0.05, Fig. 1B, C, D). However, the *METTL14* mRNA expression was evidently up-regulated in the NSCLC tissues compared to the para-NSCLC tissues (*P* < 0.05, Fig. 1E). After that, western blot and IHC staining were used to further determine the expression of METTL14 in the clinical tissues. As shown in the Fig. 1F and G, we found that the expression of METTL14 was markedly higher in the NSCLC tissues than that in the para-NSCLC tissues (*P* < 0.05). These indicated that there were higher levels of m6A methylation in the NSCLC, as well as METTL14 could be chosen as the primary subject for subsequent experiments.

### Expression of lncRNAs and their m6A methylation levels in clinical tissue samples

**Fig. 2:**
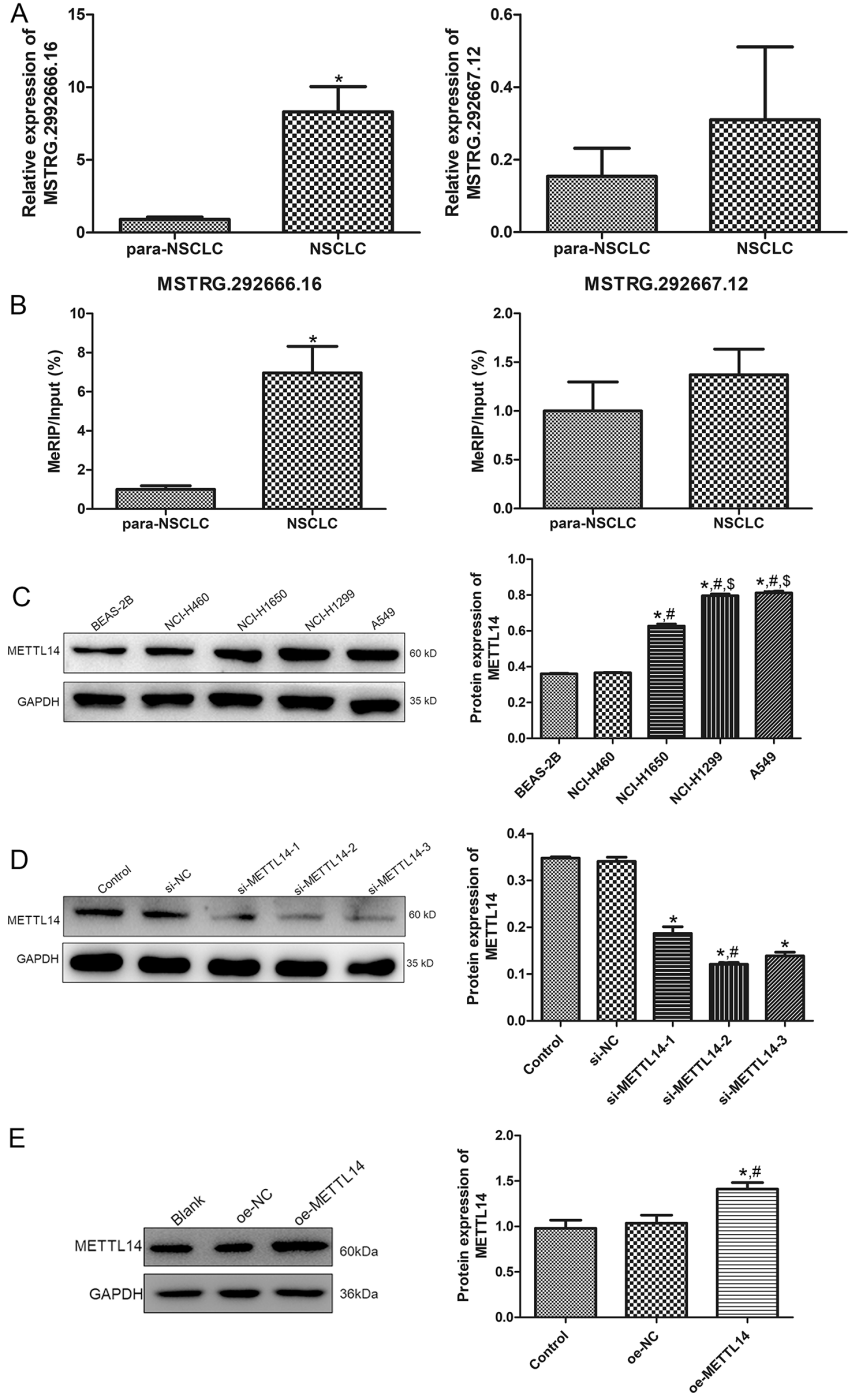
Expression of lncRNAs and their m6A methylation levels in clinical tissue samples, and METTL14 protein expression. (**A**) The expression of lncRNAs MSTRG.292666.16 and MSTRG.292667.12 in NSCLC. * *P* < 0.05, compared with the para-NSCLC. (**B**) The m6A methylation levels of lncRNAs MSTRG.292666.16 and MSTRG.292667.12 in NSCLC. * *P* < 0.05, compared with the para-NSCLC. (**C**) The protein expression of METTL14 in the different cell lines by western blot. * *P* < 0.05, compared with the BEAS-2B cells; ^#^*P* < 0.05, compared with the NCI-H460 cells; ^$^*P* < 0.05, compared with the NCI-H1650 cells. (**D**) The cell transfection efficiency after transfected with si-METTL14 by determining the expression of METTL14. * *P* < 0.05, compared with the control cells; ^#^*P* < 0.05, compared with the si-METTL14 cells. (**E**) The cell transfection efficiency after transfected with oe-METTL14 by determining the expression of METTL14. * *P* < 0.05, compared with the control cells; ^#^*P* < 0.05, compared with the oe-METTL14 cells

Our previous study showed that lncRNAs MSTRG.292666.16 and MSTRG.292667.12 were the two important factors for osimertinib sensitivity or resistance in NSCLC [19]. Therefore, we examined the expression of the two lncRNAs and their m6A methylation levels in the clinical tissue samples. Compared with the para-NSCLC tissues, the expression and the m6A methylation level of lncRNA MSTRG.292666.16 was significantly elevated in the NSCLC tissues (*P* < 0.05); while no significant difference was found in the expression and m6A methylation level of lncRNA MSTRG.292667.12 between the para-NSCLC tissues and NSCLC tissues (*P* > 0.05, Fig. 2A, B). Therefore, in the subsequent study, lncRNA MSTRG.292666.16 was selected.

### Screening of optimal NSCLC cells, and cell transfection efficiency

Due to the multiple types of NSCLC cell lines, the METTL14 expression was determined in the different NSCLC cell lines to screen the optimal NSCLC cell line. Western blot results showed that compared with the BEAS-2B cells (normal cells), the protein expression of MELLT14 was all significantly up-regulated in the cell lines of NCI-H1650, NCI-H1299, and A549 cells (*P* < 0.05), and its expression in the A549 cells was the highest among all the NSCLC cell lines (Fig. 2C). Hence, A549 cells were screened to investigate the underlying roles of METTL14 in NSCLC in vitro and in vivo.

The A549 cells transfected with si-METTL14-1/2/3 or oe-METTL14 were used to construct the cells with METTL14 knockdown or overexpression, and the cell transfection efficiency was evaluated by western blot. After transfection, si-NC/ oe-NC had no significant influence in the METTL14 expression in comparison with the control cells (*P* > 0.05, Fig. 2D, E). However, the protein expression of METTL14 was evidently down-regulated after transfected with si-METTL14-1/2/3 compared to the control cells (*P* < 0.05), and the action of si-METTL14-2 was more significant (*P* < 0.05, Fig. 2D). The METTL14 protein was significantly up-regulated in the cells with oe-METTL14 compared with the control cells (*P* < 0.05, Fig. 2E). Consequently, si-METTL14-2, and oe-METTL14 were chosen to establish the A549 cells with METTL14 knockdown and overexpression.

### Effects of METTL14 on the viability, apoptosis, and cell colony formation of A549 cells

**Fig. 3:**
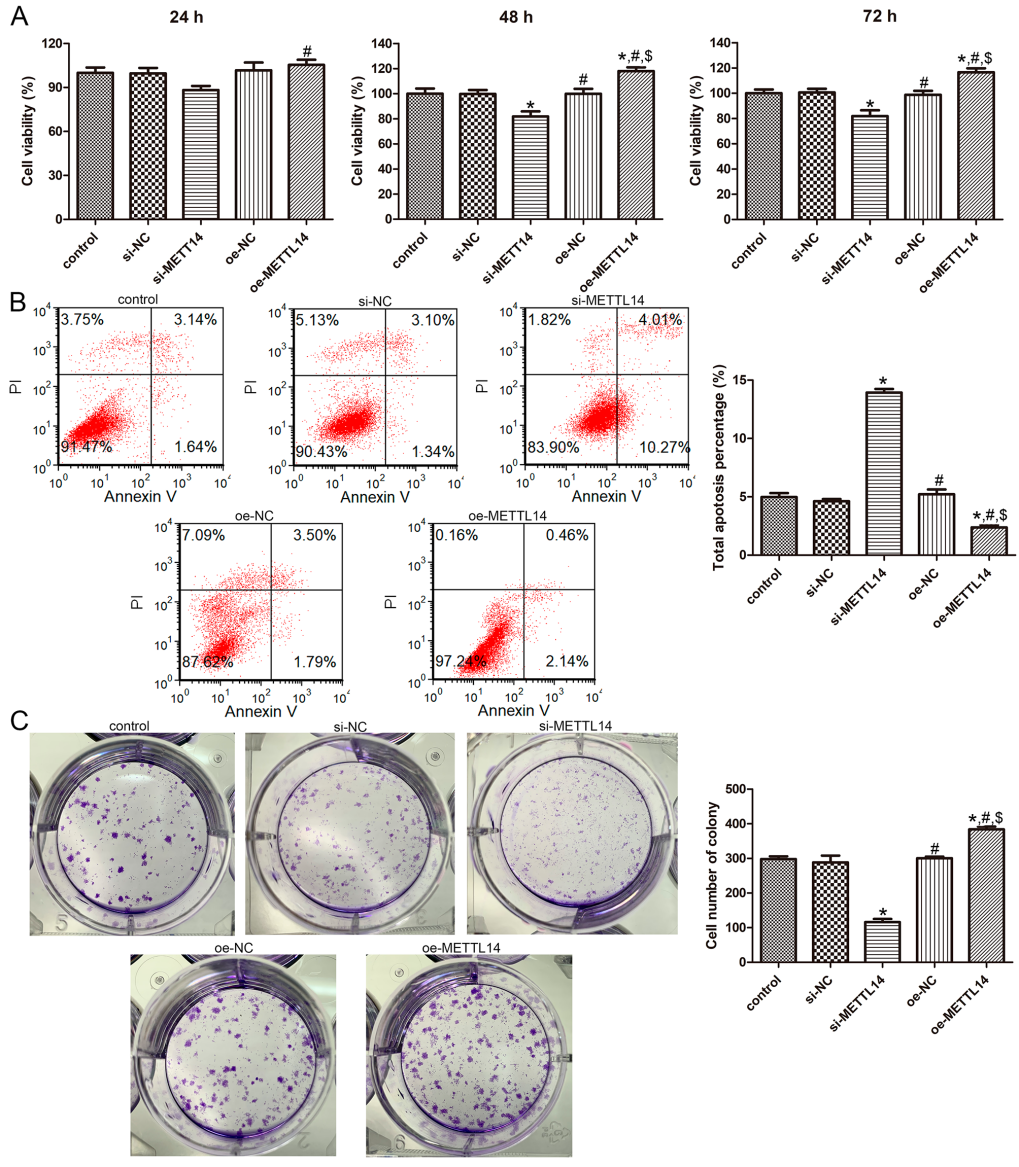
Effects of METTL14 on the viability, apoptosis, and cell colony formation of A549 cells. (**A**) The viability of A549 cells with different transfection after cultured for 24 h, 48 h, and 72 h using cell counting kit-8. (**B**) Flow cytometry used to determine the apoptosis of A549 cells with different treatments. (**C**) The cell number of colony in the A549 cells with different transfections. * *P* < 0.05, compared with the control cells; ^#^*P* < 0.05, compared with the si-METTL14 cells; ^$^ compared with the oe-NC cells

Further to explore the effects of METTL14 on the growth of A549 cells, the cell viability, apoptosis, and cell colony formation were carried out. It was obvious that no significant differences in the cell viability, apoptosis, and colony formation were observed among the control, si-NC and oe-NC groups (*P* > 0.05, Fig. 3); After cultured for 24 h, different transfection methods did not significantly change the cell viability of A549 cells relative to the control cells (*P* > 0.05, Fig. 3A). However, after 48 and 72 h of culture, METTL14 knockdown significantly reduced the viability of A549 cells, whereas its overexpression remarkedly increased the viability of A549 cells (*P* < 0.05, Fig. 3A). In addition, flow cytometry results showed that compared with the control cells, the cell apoptosis was significantly increased by si-METTL14 (*P* < 0.05), while evidently decreased by oe-METTL14 (*P* < 0.05, Fig. 3B). For cell colony formation, the cell number in the control, si-METTL14, and oe-METTL14 groups was 298 ± 11.31, 116 ± 12.73, and 384 ± 11.31, which indicated that METTL14 knockdown significantly decreased the colony formation ability of A549 cells (*P* < 0.05), whereas its overexpression remarkedly increased their ability (*P* < 0.05, Fig. 3C). These results indicated that the METTL14 silencing could inhibit the viability and colony formation of A549 cells, while promote cell apoptosis; but its overexpression could have the opposite effects.

### Effects of METTL14 on the migration and invasion of A549 cells

**Fig. 4:**
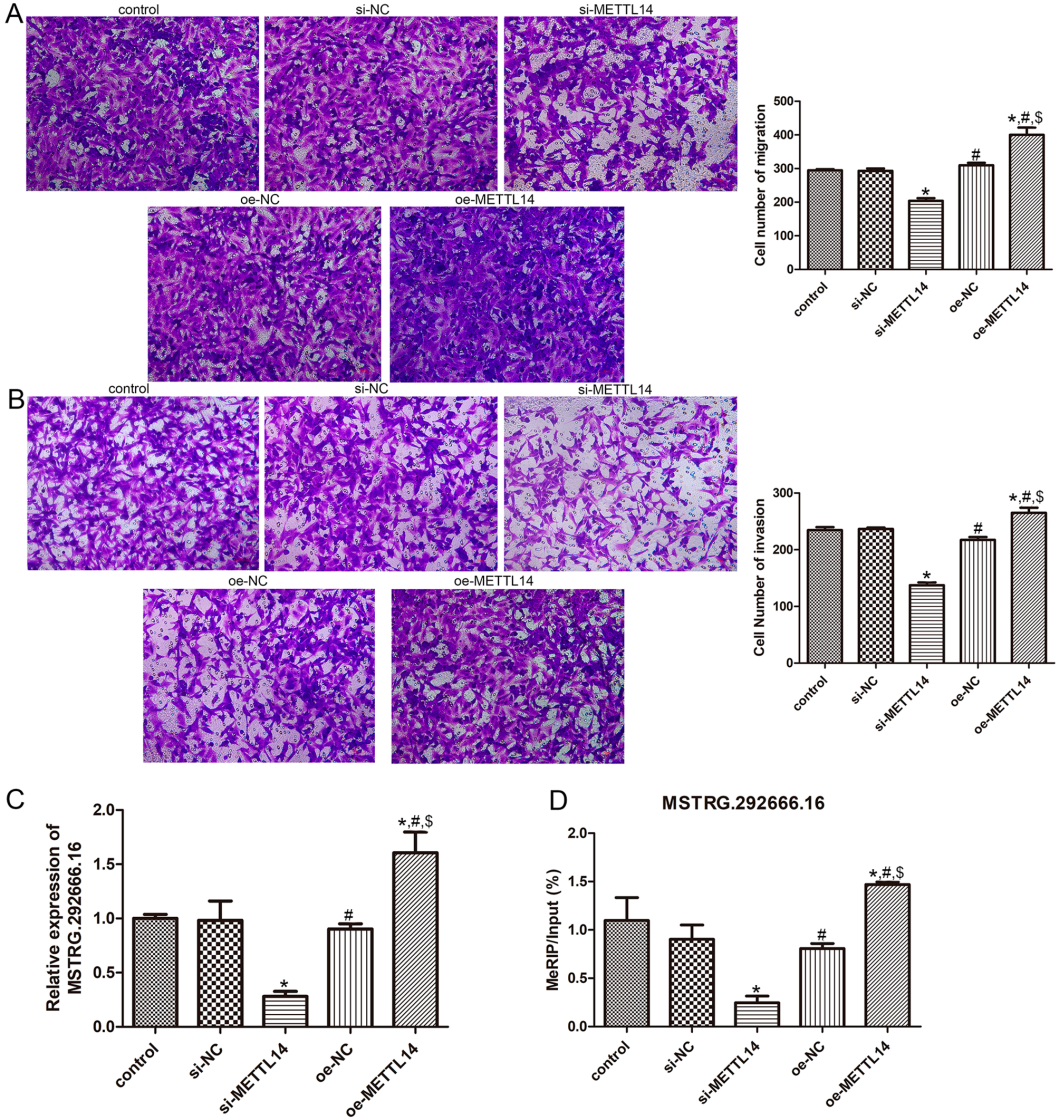
Effects of METTL14 on the migration and invasion of A549 cells, and the relationship between METTL14 and lncRNA MSTRG.292666.16. (**A**) The cell number of migration in the A549 cells with different transfections by Transwell. (**B**) The cell number of invasion in the A549 cells with different transfections by Transwell. (**C**) The expression of MSTRG.292666.16 in the different A549 cells. (D) The m6A methylation level of MSTRG.292666.16 in the A549 cells with different transfection. * *P* < 0.05, compared with the control cells; ^#^*P* < 0.05, compared with the si-METTL14 cells; ^$^ compared with the oe-NC cells

Then, Transwell assay was used to determine the migration and invasion of A549 cells. It was found there were no significant differences in the cell number of migration and invasion among the control, si-NC, and oe-NC groups (*P* > 0.05, Fig. 4A, B). Relative to the control group, the cell number of migration and invasion was both significantly reduced in the si-METTL14 group (*P* < 0.05), while was both markedly increased in the oe-METTL14 group (*P* < 0.05, Fig. 4A, B). These suggested that METTL14 knockdown could suppress the migration and invasion of A549 cells, and vice versa.

### Effects of METTL14 on the lncRNA expression and its m6A methylation level

To unearth the relationship between METTL14 and lncRNA MSTRG.292666.16, the expression and m6A methylation level of MSTRG.292666.16 were measured. In comparison with the control cells, the expression of MSTRG.292666.16 was significantly down-regulated in the cells that transfected with si-METTL14 (*P* < 0.05), whereas was obviously up-regulated in the A549 cells with METTL14 overexpression (*P* < 0.05, Fig. 4C). Additionally, the tendency of the m6A methylation levels of MSTRG.292666.16 in the cells with different transfection was similar with that of the MSTRG.292666.16 expression in the different cells (Fig. 4D).

### Effects of METTL14 on the tumor growth in vivo

**Fig. 5:**
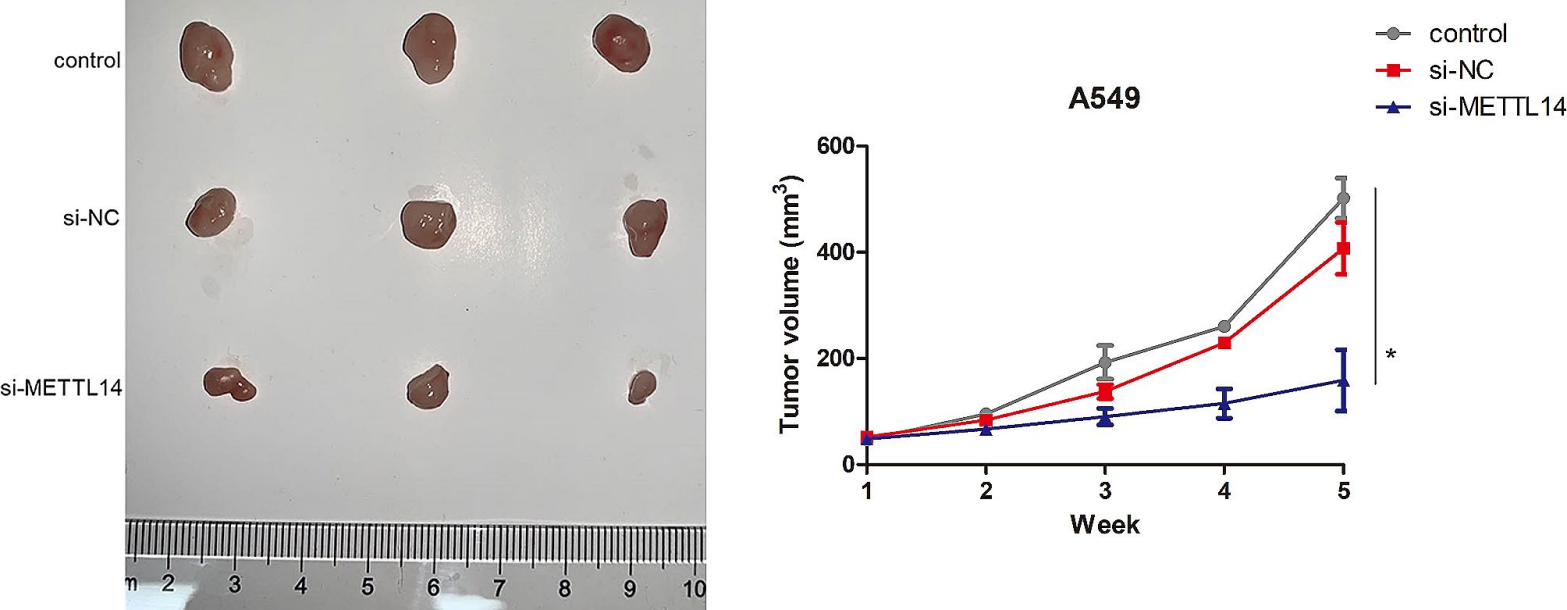
Effects of METTL14 on the tumor growth in vivo. The volume of the tumor increased over time, but METTL14 knockdown significantly reduced the volume of the tumor at week 5. * *P* < 0.05

In vivo experiments showed that the volume of the tumor increased over time, but METTL14 knockdown significantly reduced the volume of the tumor at week 5 (*P* < 0.05, Fig. 5), which implied that METTL14 knockdown could inhibit tumor formation and growth.

## Discussion

NSCLC remains the leading cause of the cancer death, and drug resistance and metastasis of lung cancer cells are important causes of the death [2]. The m6A modification has been confirmed to be closely related to the occurrence and development of tumors [21], but its specific mechanisms in NSCLC are still unknown. In this study, we found that the m6A level in the NSCLC tissues was higher, and the expression of METTL14 was also up-regulated in the NSCLC tissues compared to the para-carcinoma tissues. Then, the A549 cells with METTL14 silencing and overexpression were successfully established, as well as it was found that METTL14 knockdown could inhibit the viability, colony formation, migration, and invasion of A549 cells, while facilitate their apoptosis; but the action of METTL14 in the growth of A549 cells was opposite. In vivo experiments also showed that METTL14 knockdown could inhibit tumor formation and growth. Additionally, the m6A level of MSTRG.292666.16 was higher in the NSCLC tissues; and after METTL14 knockdown, the expression and m6A level of MSTRG.292666.16 were both significantly reduced in A549 cells, and vice versa.

There is growing evidence that m6A modifications play an integral role in inflammation, innate immunity, and antitumor effects through interactions with a variety of m6A regulators. Shen et al. [22] identified three distinct m6A clusters in triple-negative breast cancer (TNBC) through bioinformatics analysis, and then constructed a specific m6A-hypoxia signature to assess risk and predict the immunotherapy response of patients, thereby enabling more accurate treatment of TNBC in the future. Another study demonstrated that *ECE2*, a prognostic biomarker of lung adenocarcinoma (LUAD), was found to have negative correlation with m6A modification-associated genes (*HNRNPC*, *IGF2BP1*, *IGF2BP3* and *RBM1*), which suggested that *ECE2* may affect the tumor progression of LUAD by influencing the methylation level of m6A [23]. Our study found that compared with the para-carcinoma tissues, the m6A level in the NSCLC tissues was significantly elevated, which was in accordance with the previous report of Xu et al. [24]. All these indicated the importance of m6A modification in NSCLC.

The m6A modification is dynamic and reversible, and participates in biological functions mainly by the regulation of “writers”, “erasers” and “readers” [21]. In cancers, m6A modification is a double-edged sword. Recent studies have shown that the regulation of m6A modification on target genes and its effects on cancer development depends on three factors: first, whether the target is an oncogene or a tumor suppressor gene; second, abnormal m6A levels in cancers are mainly determined by the expression and activity of “writers” and “erasers”; third, target mRNAs are regulated after modification, and mainly determined by the “readers” [21, 25]. METTL14, a member of “writers” in m6A modification, can enhance the activity of methyltransferase by recognizing RNA and methyl localization, and its mutation can reduce the catalytic activity and substrate specificity of methyltransferase, resulting in reversal of methylation efficiency of consensus GGACU and non-consensus GGAUU sequences, leading to the changes in m6A levels, thus contributing to cancer occurrence and tumor metastasis [26, 27]. A previous research has reported that METTL14 level was declined in the gastric cancer tissues, and METTL14-mediated m6A modification of circORC5 could inhibit the development of gastric cancer via regulating miR-30c-2-3p/AKT1S1 [28]. Li et al. [29] manifested that METTL14 was up-regulated in the oral squamous cell carcinoma tissues and cells, as well as METTL14 could induce m6A modifictaion of lncRNA MALAT1 to up-regulate MALAT1, thus playing important roles in oral squamous cell carcinoma. Similarly, in this study, we also found that METTL14 was up-regulated in the NSCLC tissues and A549 cells, and its silencing could suppress the growth, migration, invasion of A549 cells, and promote apoptosis, thereby inhibiting the growth and development of NSCLC tumors. Chen et al. [30] illustrated that METTL14 down-regulation was associated with poor prognosis in patients with colorectal cancer (CRC); as well as METTL14 knockdown could significantly enhance the proliferation and invasion ability of CRC cells in vitro, and promote tumorigenicity and metastasis in vivo through regulating the expression and m6A level of *SOX4*. Another study of Wang et al. [31] reported that m6A levels were elevated in about 70% of pancreatic cancer samples, and METTL14 was the main enzyme that regulated m6A methylation. Additionally, it was also reported that overexpression of METTL14 could significantly promote the proliferation and metastasis of pancreatic cancer cells in vitro and in vivo via targeting PERP in an m6A-dependent manner. Combined with our results, it can be inferred that METTL14 may be the key regulator for m6A modification in NSCLC, and MTTL14 silencing may repress the growth and metastasis of NSCLC through m6A modification.

In addition, m6A modification does not only regulates occur in mRNA, but also found in a variety of non-coding RNAs, including lncRNAs, microRNAs (miRNAs), circle RNAs (circRNAs) [32]. A previous study found that METTL14 was down-regulated in the clear cell renal cell carcinoma (ccRCC) tissues, and the m6A level of Lnc-LSG1 could be regulated by METTL14, thereby playing important roles in ccRCC progression [33]. Another research manifested that the m6A levels of total RNA were increased, and METTL14 was the main m6A-related enzyme in the bladder cancer (BC); as well as METTL14-mediated m6A modification could promote malignant progression of BC through up-regulating lncDBET expression [34]. These indicated that METTL14 not only has effects on mRNA stability and translation, but also on non-coding RNAs such as lncRNAs. LncRNA MSTRG.292666.16 was found to be closely associated with Osimertinib resistance in NSCLC [19]. Exosomes derived from M2 type tumor-associated macrophages could promote Osimertinib resistance in NSCLC via the MSTRG.292666.16-miR-6836-5p-MAPK8IP3 axis [35]. Our results showed that the expression, and m6A level of MSTRG.292666.16 was increased in NSCLC; while when METTL14 knocked down, their levels were both decreased in A549 cells. Zhang et al. [36] discovered that lncRNA AC026356.1, as a downstream target of METTL14/IGF2BP2, its silencing could block the carcinogenicity of lung cancer stem-like cells, and METTL14/IGF2BP2 could regulate m6A modification and stabilization of AC026356.1. Taken together, we can speculate that lncRNA MSTRG.292666.16 may be the downstream target of METTL14, as well as METTL14 may be involved in the NSCLC development and metastasis via modulating the expression and m6A level of MSTRG.292666.16.

In conclusion, the m6A level was elevated in NSCLC, and METTL14 may be the key regulator for m6A modification. In addition, METTL14 down-regulation could inhibit the growth, migration, and invasion of A549 cells, as well as suppress the formation and development of tumors. The possible mechanism is that METTL14 plays roles in NSCLC through regulating the expression of MSTRG.292666.16 and its m6A methylation. Our findings clarify the significance of m6A modification in NSCLC, and provide an important theoretical basis and foundation for the application of METTL14 and MSTRG.292666.16 as the potential therapeutic targets or pathways in treatment of NSCLC.

## Data Availability

The dataset used and/or analyzed during the current study are available from the corresponding author on reasonable request.
